# Cessation of grazing causes biodiversity loss and homogenization of soil food webs

**DOI:** 10.1098/rspb.2023.1345

**Published:** 2023-11-15

**Authors:** Maarten Schrama, Casper W. Quist, G. Arjen de Groot, Ellen Cieraad, Deborah Ashworth, Ivo Laros, Lars Hestbjerg Hansen, Jonathan Leff, Noah Fierer, Richard D. Bardgett

**Affiliations:** ^1^ Institute of Environmental Sciences, Leiden Universiteit, Einsteinweg 2, 2333CC Leiden, The Netherlands; ^2^ Department of Earth and Environmental Sciences, The University of Manchester, Michael Smith Building, Oxford Road, Manchester M13 9PT, UK; ^3^ Biosystematics group, Wageningen UR, Droevendaalse steeg 1, 6708PB Wageningen, The Netherlands; ^4^ Laboratory of Nematology, Wageningen UR, Droevendaalse steeg 1, 6708PB Wageningen, The Netherlands; ^5^ Wageningen Environmental Research (Alterra), Wageningen UR, Wageningen, The Netherlands; ^6^ Te Pukenga–Nelson Marlborough Institute of Technology, 322 Hardy Street, Nelson 7010, New Zealand; ^7^ Environmental Microbiology and Biotechnology, Aarhus University, Frederiksborgvej 399, 4000 Roskilde, Denmark; ^8^ Department of Plant and Environmental Sciences, University of Copenhagen, Thorvaldsensvej 40, 1871 Frederiksberg, Denmark; ^9^ Cooperative Institute for Research in Environmental Sciences, University of Colorado, Boulder, CO 80309, USA; ^10^ Department of Ecology and Evolutionary Biology, University of Colorado, Boulder, CO 80309, USA

**Keywords:** soil fauna, grazing, soil communities, land abandonment, biotic homogenization, *α*-diversity

## Abstract

There is widespread concern that cessation of grazing in historically grazed ecosystems is causing biotic homogenization and biodiversity loss. We used 12 montane grassland sites along an 800 km north–south gradient across the UK, to test whether cessation of grazing affects local *α*- and *β*-diversity of below-ground food webs. We show cessation of grazing leads to strongly decreased *α*-diversity of most groups of soil microbes and fauna, particularly of relatively rare taxa. By contrast, the *β*-diversity varied between groups of soil organisms. While most soil microbial communities exhibited increased homogenization after cessation of grazing, we observed decreased homogenization for soil fauna after cessation of grazing. Overall, our results indicate that exclusion of domesticated herbivores from historically grazed montane grasslands has far-ranging negative consequences for diversity of below-ground food webs. This underscores the importance of grazers for maintaining the diversity of below-ground communities, which play a central role in ecosystem functioning.

## 1. Introduction

The cessation of grazing is a common feature of the European landscape and is expected to rise sharply over the next decade [1], especially in low-productivity, mountainous areas where previously extensively grazed lands are increasingly being taken out of agricultural production [1–4]. Extensively managed, semi-natural grasslands are widespread across Europe, often grazed since Roman or even pre-Roman times [5,6], and support an important component of regional biodiversity, delivering multiple ecosystem functions and services [7,8]. This has resulted in grassland ecosystems with spatially heterogeneous vegetation [9]. Based on studies focused on plants, there is widespread concern that the cessation of grazing in these ecosystems is causing biotic homogenization due to a loss of rare specialist species and an increase in common generalists, as well as overall declines in plant biodiversity [10–13]. Further, biotic homogenization and associated loss of biodiversity resulting from grazer exclusion is likely to impact ecosystem functioning [13–15].

Despite the prevalence of cessation of grazing from historically grazed ecosystems, major uncertainties exist regarding its impact on biodiversity and ecosystem functioning. One particular uncertainty concerns its impact on different components of biodiversity, which have been observed to operate independently of each other. Species richness at the local, plot scale (*α*-diversity) is probably driven by changes in land management [16], whereas compositional (between plot) variation (*β*-diversity), which includes variation in the taxonomic composition of communities across sites, is driven by a range of factors that operate from small to larger scales [17]. Therefore, while *α*-diversity may be stable or increasing in some areas, *β*-diversity could be decreasing due to biotic homogenization, i.e. communities from different sites become more similar in composition [18–20]. There is mounting evidence that the cessation of livestock grazing influences these different attributes of biotic homogenization of above-ground communities, including plants [11,20,21] and insects [22,23], but far less is known regarding the effects on communities of below-ground organisms. Soil biodiversity regulates a number of key ecosystem functions and services, for instance organic matter decomposition, plant nutrient availability, nutrient leaching, and soil structural stability [24–27]. While some studies have examined the effects of cessation of livestock grazing on below-ground communities in grasslands [13,28], these studies generally focused on specific groups of soil organisms (but see [12,29,30]), short time spans since grazing removal [12] or a narrow range of climate and soil conditions [30]. Given this, there is a clear need for an improved understanding of the long-term impact of the cessation of grazing on the composition and diversity of below-ground communities.

**Figure 1.**
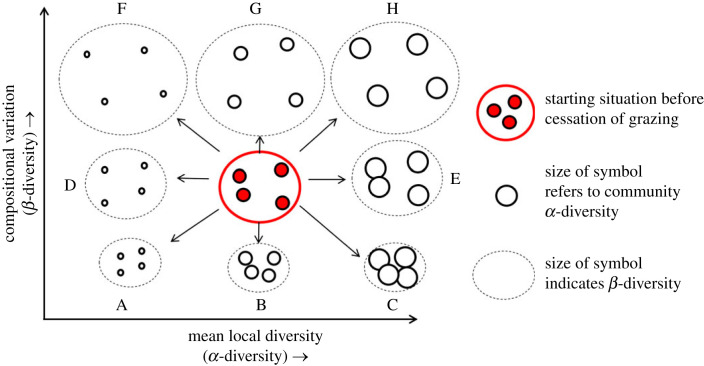
Potential changes in *α-* and *β*-diversity of soil organisms resulting from the cessation of grazing. *α*-diversity can potentially increase or decrease independently of changes in *β*-diversity, which can also increase or decrease, from a starting position (indicated in red).

Here, we explore how cessation of grazing impacts *α*- and *β*-diversity of both plants and below-ground communities, by analysing resulting changes in vegetation and a wide range of soil faunal and microbial groups. We use the term ‘cessation of grazing’ to represent the termination of grazing by domesticated herbivores, such as cattle or sheep, but not the intentional removal of naturally occurring grazers such as hare, deer and voles. We used a series of 12 montane grassland sites positioned along an 800 km north–south gradient of the UK and covering several of the UK's main montane grassland regions, each with several paired plots that were either subject to historical grazing by sheep or had livestock grazers excluded by fencing for 10–65 years. We focused on montane grasslands because they are a prominent feature of the European landscape and have been extensively grazed by sheep for centuries, forming the backbone of the sheep farming industry across Europe [31]. Further, the cessation of livestock grazing is commonplace in mountain regions of Europe, including the UK, and is recognized as a key aspect of land abandonment [1] and rewilding [32], with the potential to have multiple, but largely unknown, effects on local diversity and compositional variation in below-ground communities among sites (figure 1; after [22]).

**Figure 2.**
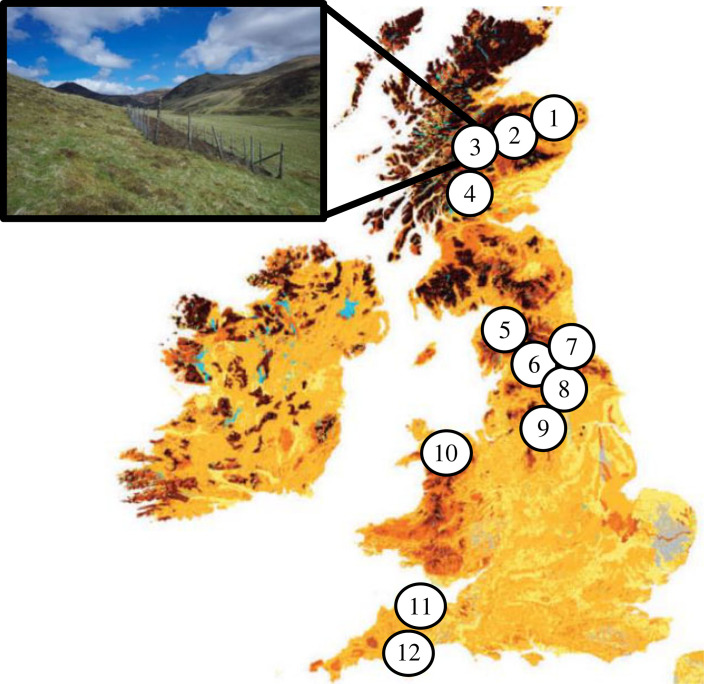
Locations of the 12 sites. Numbers correspond to the different sites: (1) Glen Saugh; (2) Ben Lawers; (3) Glen Finglas; (4) Glen Shee; (5) Lake District; (6) Moor House; (7) North Pennines; (8) Yorkshire Dales; (9) Peak District; (10) Snowdonia; (11) Exmoor; (12) Dartmoor. Background map depicts soil organic matter concentrations [33]: darker colours indicate high carbon stocks. Picture of Glen Shee (inset) shows a typical pattern as a result of grazing: higher grass cover at the grazed side of the fence with dominance of *Nardus stricta* and dominance of ericaceous shrubs, such as *Calluna vulgaris* on the side of the fence where grazers were excluded. Photo: M. Schrama.

**Figure 3.**
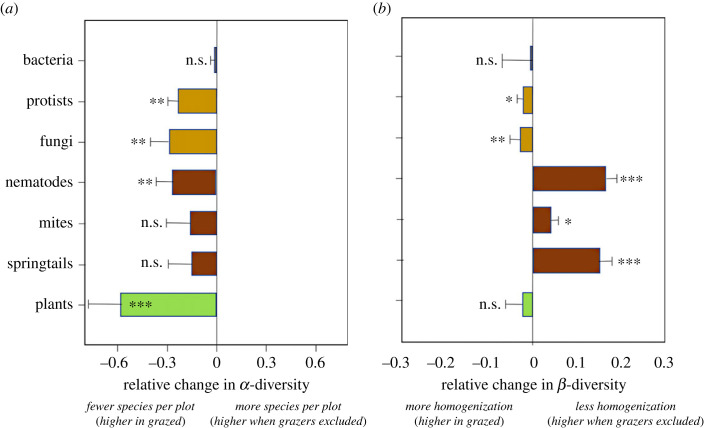
Response ratios of different species groups to cessation of grazing (±s.d.). Light brown bars indicate soil microbes, dark brown bars indicate soil fauna, green bars indicate plants. (*a*) Effects on *α*-diversity (species richness). (*b*) Effects on *β*-diversity (homogenization). Positive values indicate an increase in diversity as a result of cessation of grazing and a negative value indicates a decrease in diversity. Asterisks indicate significant differences: *** *p* < 0.001; ** 0.001 < *p* < 0.01; *0.01 < *p* < 0.05; n.s., not significant.

**Figure 4.**
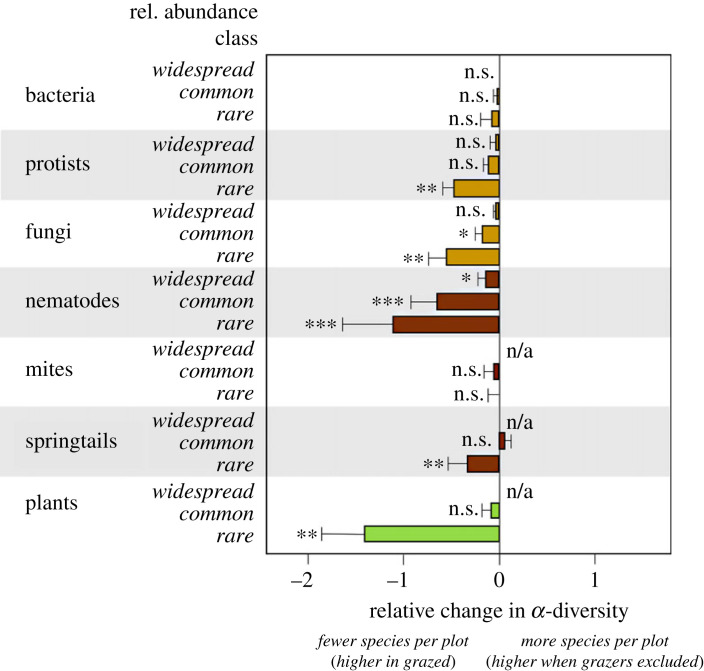
Response ratios of *α*-diversity (± s.d.) of relatively rare, common and widespread microbes, plant species and soil fauna to cessation of grazing. Light brown bars indicate soil microbes, dark brown bars indicate soil fauna, green bars indicate plants. A positive value indicates an increase in species richness in response to cessation of grazing; a negative value indicates a decrease in species richness as a result of cessation of grazing. Asterisks indicate significant differences: *** *p* < 0.001; ** 0.001 < *p* < 0.01; *0.01 < *p* < 0.05; n.s., not significant.

Changes in *α*- and *β*-diversity can occur simultaneously and have positive, neutral or negative relationships. As such, we tested a range of hypotheses, namely that: (1) increased *α*-(plot-based) diversity occurs when cessation of grazing results in a higher degree of local environmental variation, and an increased availability of niches supports more species (scenarios C, E and H in figure 1); (2) decreased *α*-diversity happens when grazer exclusion results in a reduction in local environmental heterogeneity, causing loss of rare species and/or gain of generalist species (scenarios A, D and F in figure 1); (3) a decrease in *β*-(site-based) diversity may occur independently of changes in *α*-diversity, which is expected when the removal of grazing has a homogenizing effect on the composition of local communities within a given area, independent of the effect on local soil community species richness (scenarios A, B and C in figure 1); (4) if current land management causes strong homogenization of soil communities, we expect that cessation of grazing will increase *β*-diversity through a gradual divergence of communities, which may happen independently of changes in *α*-diversity by changing communities in various directions through differential species losses and gains (scenarios F, G and H in figure 1). We tested these hypotheses using our unique dataset of different components of the below-ground food web from long-term paired grazed and ungrazed exclosures across the UK.

## 2. Methods

### (a) Site description

We selected 12 montane grassland sites across an 800 km north–south gradient of the UK (figure 1). Sites were selected based on the following criteria: (1) grasslands had never received inorganic fertilizers or herbicides; (2) grazer exclusion plots had to be present for at least 10 years; (3) sites needed to be sufficiently far apart (greater than 10 km) to be considered independent from each other; and (4) the main form of historical management at the site is extensive grazing by sheep. Sites were typically grazed extensively by pure bred sheep at stocking densities of 1–2 ewes per hectare per year, although historical variation in grazing pressure across sites has resulted in mosaics of vegetation with patches of short and tall grass, interspersed with patches of dwarf-shrubs dominated by *Calluna vulgaris*, *Vaccinium myrtillus*, *Erica tetralix* and *Erica cinerea*. All sites were visited and sampled once, between 28 April and 7 June 2015. Fenced grazing exclosures varied in size from 25 m^2^ to 10.66 ha and in age since cessation of grazing from 10 to 65 years (table 1; electronic supplementary material, table S1; figure 2). While wild herbivores, especially deer, may have accessed some of the exclosures, albeit at very low densities, no active re-introductions of wild herbivores had taken place at any of the sites. At the sites, we sampled four paired 5 × 5 m plots where extensive grazing had been excluded by fencing and four adjacent grazed plots (figure 2). At one location, Exmoor (site 11, figure 2), we sampled three paired sites, and in the Peak District (site 9, figure 2), we sampled six sites; therefore, the total number of plots was 98, half grazed and half ungrazed, from 12 distinct sites. The elevation from the sites varied between 300 m and 700 m asl (table 1), and differed in underlying geology, climatic conditions, soil characteristics and dominant plant species (electronic supplementary material, table S1–S4). In each plot, we assessed the vegetation composition and biomass (see electronic supplementary material for details). Soil samples were collected to determine soil abiotic properties (bulk density, water content, carbon (C), nitrogen (N), phosphorus (P) content, pH and the potential nitrogen-mineralization; for further details, see electronic supplementary material, methods S6.

**Table 1.** Site details for all 12 locations; site numbers correspond to figure 2.

site name	cessation of grazing since	GPS (N)	GPS (W)	altitude (m asl)
(1) Glen Saugh	1980	56°54'04.3″ N	2°33'09.0″ W	329
(2) Ben Lawers	1991	56°32'26.5″ N	4°09'10.8″ W	578
(3) Glen Flinglas	2006	56°16'36.1″ N	4°27'00.7″ W	392
(4) Glen Shee	1990	56°51'16.2″ N	3°25'41.5″ W	558
(5) Lake District	1990	54°39'33.1″ N	3°10'57.0″ W	492
(6) Moor House	1957	54°40'59.5″ N	2°27'00.0″ W	684
(7) North Pennines	1965	54°48'01.8″ N	2°20'02.4″ W	481
(8) Yorkshire Dales	2000	54°11'38.4″ N	2°20'59.3″ W	350
(9) Peak District	1995	53°22'47.3″ N	1°40'52.7″ W	435
(10) Snowdonia	1950	53°09'46.1″ N	3°57'49.0″ W	665
(11) Exmoor	1998	51°03'32.8″ N	3°41'09.6″ W	303
(12) Dartmoor	2006	50°26'23.6″ N	3°54'36.4″ W	347

### (b) Assessment of the composition of the below-ground community

#### (i) Nematode communities

Nematodes were extracted from 200 g of composite soil sample using the elutriator—cotton wool filter method [34]. Nematode suspensions were concentrated, and DNA was extracted by a lysis buffer including mammalian DNA as an external standard to monitor losses due to sample handling and DNA purification [35]. DNA extracts were purified using a glass-fibre column-based procedure [36]. Purified DNA extracts were stored at −20°C. To assess overall nematode biodiversity across all sites, 5 µl aliquots of all purified extracts were combined. The resulting mixture was analysed by qPCR using 72 nematode taxon-specific primer sets (electronic supplementary material, table S5). Based on the outcome of the overall biodiversity assessment, 30 nematode taxa were selected for qPCR-based quantification in each of the 98 samples. Two additional qPCR primer sets were used: one primer set was used to assess total nematode densities per sample, and a second primer set was used to quantify DNA levels of the external standard. Quantitative PCR reactions were executed and Ct-values were converted to nematode counts per 200 g soil. For details, see Vervoort, Vonk [35], Quist *et al*. [37].

#### (ii) Microbial communities

Genomic DNA of bacteria, fungi, and protists was extracted from 1.5 g of each composite soil sample using the Qiagen DNeasy PowerSoil 96-well extraction method. DNA was amplified in triplicate using primers specific to targeted regions within either the 16S or 18S rRNA gene (for prokaryotic and eukaryotic analyses, respectively). A portion of the 16S rRNA gene was amplified using the archaeal- and bacterial-specific primer set 515f/806r [38]. This 16S rRNA gene primer set is designed to amplify the V4–V5 region of both Archaea and Bacteria, has few biases against specific taxa and accurately represents phylogenetic and taxonomic assignment of sequences [39]. The 18S rRNA gene was amplified using the eukaryotic-specific primer set F1391 (50-GTACACCGCCCGTC-30) and REukBr (50-TGATCCTTCTGCAGGTTCACCTAC-30). The 18S rRNA gene primer set is designed to amplify the hypervariable V9-region of eukaryotes, with a focus on microbial eukaryotic lineages [40], including both protists and fungi. Amplicons were sequenced on two lanes of a 2 × 151 bp sequencing run on the Illumina HiSeq 2500 operating in Rapid Run Mode, following [41].

#### (iii) Microarthropod communities

To determine the community composition of microarthropods (mites: Acari, and springtails: Collembola), the large intact soil core was extracted using the Tullgren extractors at Lancaster University. Per plot, the batch of extracted microarthropods was collected in 96% ethanol and further processed to enable DNA-based identification. DNA extraction was performed using the DNeasy Blood & Tissue kit. DNA was amplified using the MiteMinBarF7 and MiteMinBarR4 primers, which target an approximately 200 bp fragment located within the cytochrome oxidase subunit 1 (COI) region, and were specifically designed to cover a wide diversity of microarthropods in NW-European grasslands [42]. At Aarhus University (Roskilde, Denmark), amplicons were prepared for in-house paired-end sequencing on an Illumina MiSeq platform, using the Nextera XT indexing kit (Illumina, San Diego, CA, USA). The resulting amplicon libraries were purified using HighPrep PCR (Magbio Genomics Inc., Gaithersburg, MD, USA) beads, quantified and equimolarly pooled, upon sequencing using the 250 bp paired-end MiSeq version 2 reagent kit (Illumina, San Diego, CA, USA).

### (c) Data analysis

#### (i) Bioinformatic processing

Bioinformatic processing of the sequence data was conducted for microarthropods (springtails and mites; COI), bacteria (16S), and fungi and protists (18S) according to standard procedures (see electronic supplementary material, methods S6).

#### (ii) Diversity calculations

Changes in species richness resulting from cessation of grazing were calculated per site (e.g. Lake District) for the different groups using the formula: Δ *α* = (*α*[ungrzaerd] – *α*[grazed])/ *α*[ungrazed], which gives a response ratio for the site where grazing was excluded. We refrained from using the Shannon's and Simpson's indices as primer choice affects the relative abundance of phylotypes [43]. Positive values represent an increase in species richness at a given site, negative values represent a decrease. To calculate *β*-diversity for each taxonomic group, we calculated the dissimilarity among all grazed and among all ungrazed plots at a given site, following Ferrier *et al.* [44]. To obtain this metric, we calculated a Bray–Curtis dissimilarity matrix within site based on presence–absence data, using the *vegan* package [45]. We then averaged the dissimilarities per site to obtain an estimate for *β*-diversity for both treatments at each of the 12 locations. For example, the *β*-diversity of the grazed treatment in the Yorkshire Dales (which had four independent plots) was based on the average dissimilarity in each soil organismal group for all pairwise combinations of grazed plots 1, 2, 3 and 4, which were subsequently averaged. We used the same procedure for the ungrazed plots. Changes in community dissimilarity between grazing/grazing removal treatments were calculated as follows: Δdissimilarity = (dissimilarity[ungrazed] – dissimilarity[grazed]) / dissimilarity[ungrazed].

To investigate the effect of cessation of grazing on the alpha diversity of relatively rare, common and widespread taxa, we performed a separate analysis. For this, we divided taxa into three groups representing the upper quartile, median quartiles and lower quartile. Taxa that were present in less than 25% of all plots (*n* = 98) were considered relatively rare (lower quartile); taxa present in 25 to 75% of all plots were considered relatively common (median quartiles); and taxa present in more than 75% of all plots were considered relatively widespread (upper quartile; hereafter referred to as rare, common and widespread). We then investigated for each of these relatively arbitrary cohorts, how species richness within each of the organismal groups responded to cessation of grazing using a similar method as explained above for *α*-diversity. We refrained from calculating *β*-diversity for the same cohorts of rare, common and widespread taxa, as *β*-diversity (a measure of differentiation between plots or sites based on the relative abundance of the various species present at the sites) requires the species in the various abundance classes to be calculated in an ecologically meaningful way. So, although theoretically possible, calculating this metric for subsets of more or less common fractions of species groups leads to results that are very difficult to interpret.

#### (iii) Statistical procedures

First, to test the effect of cessation of grazing on the response ratios of *α-* and *β*-diversity on each of the species groups, Gaussian linear mixed models were used, with grazing/grazing removal treatment as a fixed predictor and site as a random effect. To test for differences among *α*-diversity of rare, abundant and widespread taxa, we used the same statistical procedure. Second, to investigate which of the biotic and abiotic properties affected *α-* and *β*-diversity, we used a separate series of linear mixed effect models for each group of soil organisms. We separately ran the models for *α*-diversity (i.e. species richness) and *β*-diversity (i.e. B-C dissimilarity) of each of the soil fauna groups, and scaled the responses prior to analysis. Out of a total of 48 plots, eight had very deep organic layers of greater than 100 cm deep and were excluded from this analysis, as we were unable to estimate the carbon stock of these sites. We included the following variables as fixed predictors: grazing/grazing removal treatment, pH, soil carbon (% dw), depth of organic layer (cm), litter biomass (g m^−2^), above-ground biomass (g m^−2^), cover of grasses & herbs, average soil bulk density (g dm^−3^), soil moisture (g dm^−3^), plant richness (number of species per 2 × 2 m quadrat), mineral N (mg kg^−1^) and two-way interactions between each of these variables and the grazing/grazing removal treatment. For each species group, we constructed a full model consisting of all fixed effects and their interaction with grazing removal. We then used an information-theoretic approach to compare the weight of evidence for each possible sub-models (*r*) using Akaike information criterion corrected for small sample size (AICc [46]). For each model *i,* we calculated the AICc difference to the top-ranked model (Δ_i_ = AICc_i_ – AICc_min_), and the Akaike model weight

$$w_{i}=\frac{\exp(-\Delta_{i}/2)}{\left(\sum_{r=1}^{R}\exp(-\Delta_{r}/2)\right)}.$$

We averaged the models with Δ_i_ < 4, using model weights to generate estimates of coefficients (AICc-weighted average of coefficient values) [46] in the MuMIn package [47]. We calculated the pseudo-*R*^2^ of each of the averaged models using r.squaredGLMM function in the MuMIn package [47]. Site was included as a random predictor throughout. In addition, we explored whether cessation of grazing affected plant species composition, by conducting a non-metric multidimensional scaling analysis, using the R package *vegan* [45].

## 3. Results

### (a) Effects of cessation of grazing on vegetation and soil characteristics

Consistent with our expectations, across all sites, cessation of grazing had strong effects on plant functional groups (electronic supplementary material, figure S1), but not on plant species composition (electronic supplementary material, figure S2). In general, cessation of grazing resulted in plant communities becoming more dwarf-shrub or fern dominated on acid soils, or dominated by tall grasses (e.g. *Deschampsia cespitosa*) on more alkaline soils. Sites where grazing was excluded had marginally higher above-ground biomass (*F*_1,73_ 3.28, *p* = 0.07), although this varied strongly by site (*F*_11,73_ 2.77, *p* = 0.004). Across sites, cessation of grazing caused the litter layer depth to increase on average by 28% or 4 cm (*F*_1,60_, *p* = 0.04). Cessation of grazing also resulted in changes in soil abiotic properties, including lower mean soil temperature (*F*_1,53_ 22.0, *p* < 0.001) and reduced electrical conductivity (*F*_1,53_ 8.8, *p* = 0.004). Grazing removal resulted in slightly higher soil inorganic nitrogen concentrations (*F*_1,69_ 4.4, *p* = 0.04), but we detected no changes in rates of potential N mineralization (*p* > 0.1) nor in pH (*p* > 0.1). Soil bulk densities varied across sites (*F*_11,88_ 8.84, *p* < 0.001), but were not affected by cessation of grazing (*p* > 0.1).

### (b) Effects of cessation of grazing on *α*-diversity of plant and soil communities

For the analysis of changes in diversity, we used a total of 113 mite and 79 springtail phylotypes, 30 nematode taxa (families/genera), 2068 protist phylotypes, and 2179 fungal and 10336 bacterial phylotypes. Above-ground, we recorded 76 species of vascular plants. Estimated species richness levels (as a measure of *α*-diversity) of soil eukaryotes, soil fauna and vascular plants were consistently reduced as a result of grazing removal (figure 3). This decline was most pronounced for vascular plants, which declined from an average of 8.0 species in managed plots to 5.8 species (response ratio: −0.57) in grazing removal plots (*F*_1,74_ 14.2, *p* < 0.001; figure 3). Nematode richness decreased with grazing removal, as illustrated by the negative response ratio of −0.27 (*F*_1, 71_ 19.4, *p* < 0.001; figure 3); species richness of mites and springtails showed a similar, but non-significant trend. Within the microbes, species richness of microbial eukaryotes declined with grazing removal: the phylotype richness of fungi decreased by 19% in the grazing removal treatment (response ratio −0.29, *F*_1, 72_ 13.6, *p* < 0.001; figure 3) and the phylotype richness of protists decreased by 17% (response ratio −0.21, *F*_1, 72_ 9.8, *p* = 0.003). By contrast, phylotype richness of bacteria did not show a significant response to grazing removal (0.4% change; figure 3).

### (c) Drivers of *α*-diversity

Overall, our models explained a relatively large amount of variance in species richness (*α*-diversity: conditional *R_c_*^2^ of the top models (ΔAIC < 4) ranged between 0.22 and 0.67). A considerable amount of this variance was explained by site, as indicated by the difference in model fit between the conditional and marginal *R*^2^ (*R_c_^2^* –*R_m_*^2^): *α*-diversity variation explained by site ranged between 0.0 and 0.36 across variables). Site was a particularly strong predictor of *α*-diversity of mites, and springtails where it explained at least three times as much variation as all other variables combined; by contrast, site hardly explained any variation in the *α*-diversity of the microbial groups (bacteria, fungi and protists, table 2). Explanatory soil variables differed strongly between groups of soil organisms (table 2). Species richness of the microbial groups (bacteria, fungi and protists) was most strongly and positively related to soil pH and to a lesser extent to litter biomass, soil bulk density and moisture content. Species richness of nematodes was also related to pH, albeit less strongly than for soil microbes. Across the top models, nematode species richness was explained by soil carbon content, and interaction effects of grazing removal with each of pH, litter biomass and above-ground biomass. Conversely, species richness of springtails and mites was unrelated to soil pH and in general poorly explained by any of the factors included.

**Table 2.** Summary of relationships between *α-* and *β*-diversity of the different soil fauna and microbial groups and abiotic drivers Values show estimated coefficient of the averaged model ± s.e. Coefficients for those models with ΔAIC < 4 were averaged, weighted by the Akaike weight of each model. Variables that were not represented in any of these models were not included in this table. In brackets, the cumulative Akaike weight for the models in which this variable occurred is provided; followed by the number of models in which this variable was present. The total number of models with ΔAIC < 4 is also presented, as is the range of marginal *R*^2^ (*R*^2^*m*, fixed effects only) and conditional *R*^2^ (*R^2^c*, fixed and random effects), and the site effect (*R*^2^*c* – *R^2^m*) for these models.

factor	drivers of *α*-diversity (estimates)						drivers of *β*-diversity					
	nematodes	springtails	mites	protozoa	bacteria	fungi	nematodes	springtails	mites	protozoa	bacteria	fungi
grazing/removal treatment	−0.33 ± 0.22 (0.96;8)	0.1 ± 0.21 (0.06;1)	0.03 ± 0.22 (0.07;1)	0.29 ± 0.2 (0.22;5)	0.13 ± 0.12 (0.08;1)		1.09 ± 0.19 (1;7)	0.59 ± 0.17 (1;12)	0.34 ± 0.2 (0.36;6)	−0.12 ± 0.16 (1;3)	−0.01 ± 0.14 (0.15;1)	−0.22 ± 0.09 (0.5;3)
pH	0.38 ± 0.21 (0.14;1)	−0.09 ± 0.14 (0.04;1)	−0.1 ± 0.15 (0.05;1)	0.47 ± 0.11 (1;15)	0.65 ± 0.1 (1;5)	0.59 ± 0.08 (1;4)	−0.28 ± 0.12 (0.38;3)	−0.32 ± 0.16 (0.4;5)		−0.12 ± 0.11 (0.12;1)		
soil carbon %	−0.36 ± 0.11 (0.44;3)	0.14 ± 0.13 (0.06;1)	−0.17 ± 0.13 (0.09;1)	−0.26 ± 0.12 (0.33;5)	−0.22 ± 0.09 (0.63;3)			0.11 ± 0.09 (0.03;1)	0.17 ± 0.12 (0.1;2)			0.06 ± 0.07 (0.08;1)
organic layer depth		0.25 ± 0.14 (0.22;2)	0.15 ± 0.15 (0.07;1)			0.1 ± 0.07 (0.1;1)		−0.13 ± 0.11 (0.03;1)			0.14 ± 0.11 (0.12;1)	
litter biomass	−0.47 ± 0.24 (0.78;7)		−0.13 ± 0.11 (0.07;1)		0.1 ± 0.06 (0.09;1)			0.46 ± 0.2 (0.07;2)	0.14 ± 0.1 (0.07;2)			
above-ground biomass	−0.32 ± 0.16 (1;9)	0.12 ± 0.12 (0.05;1)	0.14 ± 0.12 (0.07;1)						0.18 ± 0.11 (0.16;3)			
cover of grass + herbs		−0.13 ± 0.12 (0.05;1)		0.29 ± 0.16 (0.11;3)			0.23 ± 0.12 (0.33;3)		0.13 ± 0.13 (0.03;1)	−0.21 ± 0.13 (1;3)		0.13 ± 0.06 (0.34;2)
bulk density		−0.2 ± 0.14 (0.09;1)	0.15 ± 0.16 (0.07;1)	0.17 ± 0.11 (0.07;2)				−0.06 ± 0.16 (0.06;1)	−0.14 ± 0.15 (0.05;1)		−0.24 ± 0.14 (0.15;1)	
soil moisture	−0.12 ± 0.12 (0.04;1)	0.11 ± 0.13 (0.04;1)	−0.13 ± 0.13 (0.06;1)	−0.27 ± 0.1 (0.67;10)	−0.14 ± 0.07 (0.09;1)	−0.25 ± 0.08 (1;4)			0.22 ± 0.12 (0.25;4)	0.11 ± 0.08 (0.11;1)		
plant richness	0.17 ± 0.11 (0.04;1)	−0.19 ± 0.12 (0.15;2)				0.11 ± 0.08 (0.1;1)	0.21 ± 0.1 (0.63;4)	−0.19 ± 0.1 (0.26;3)			−0.12 ± 0.09 (0.11;1)	
mineral N			0.14 ± 0.12 (0.07;1)	0.21 ± 0.09 (0.54;8)		0.1 ± 0.08 (0.1;1)		−0.13 ± 0.08 (0.04;1)	0.16 ± 0.11 (0.07;2)			
cessation of grazing × pH	−0.42 ± 0.18 (0.12;2)							0.35 ± 0.15 (0.26;3)				
cessation of grazing × soil carbon%												−0.25 ± 0.09 (0.08;1)
cessation of grazing × litter biomass	0.73 ± 0.26 (0.44;3)							−0.53 ± 0.2 (0.06;1)				
cessation of grazing × abovegr. biom.	−0.34 ± 0.22 (0.16;3)											
Cessation of grazing × cover grass + herbs				−0.35 ± 0.17 (0.03;1)						0.7 ± 0.16 (1;3)		
Cessation of grazing × bulk density								−0.32 ± 0.14 (0.03;1)			0.46 ± 0.14 (0.15;1)	
**number of models with ΔAIC < 4**	9	11	10	15	5	4	7	12	15	3	4	5
**marginal *R*^2^ (range)**	0.28–0.37	0–0.08	0–0.02	0.42–0.50	0.60–0.64	0.52–0.54	0.22–0.29	0.10–0.19	0–0.07	0.14–0.16	0–0.05	0–0.03
**conditional *R*^2^ (range)**	0.50–0.58	0.21–0.28	0.28 −0.38	0.47–0.55	0.63–0.67	0.54–0.55	0.68–0.76	0.66–0.74	0.43–0.51	0.69–0.70	0.61–0.67	0.85–0.87
**site effect (range)**	0.18–0.22	0.18–0.27	0.26 0.36	0.03 0.07	0.02–0.03	0–0.02	0.44–0.49	0.53–0.60	0.40–0.47	0.53–0.55	0.61–0.64	0.83–0.85

### (d) Effects of cessation of grazing on *β*-diversity

Cessation of grazing caused a significant decrease in *β*-diversity for protists and fungi (both approx. 5%), whereas *β*-diversity of plants and bacteria was unaffected (figure 3*b*). By contrast, cessation of grazing caused an increase in *β*-diversity for mites (5%), springtails (15%) and nematodes (15%).

### (e) Drivers of *β*-diversity

Overall, models explained a relatively large amount of variance in *β*-diversity: mean *R_c_*^2^ = 0.68, range 0.44–0.87; table 2). Across all groups of soil organisms, most variance was explained by site, as indicated by the difference in model fit between the conditional and marginal *R*^2^ (mean *R_c_*^2^ –*R_m_*^2^ = 0.36, range 0.44–0.84; table 2). Of the fixed effects, cessation of grazing was the main factor responsible for differences in *β*-diversity: grazing removal had a positive impact on *β*-diversity of soil fauna groups and a negative effect on *β*-diversity of protozoa, bacteria and fungi (table 2), much in line with the results on the response ratios (figure 3*b*). Moreover, soil pH was an important explanatory variable for *β*-diversity of nematodes and springtails, and above-ground biomass and soil moisture were important explanatory variables for *β*-diversity of mites but neither of these variables was important for any of the soil microbial groups.

### (f) Effects on of cessation of grazing on rare, common and widespread taxa

For plants, springtails and mites, no taxa were classified as ‘relatively widespread’. In general, cessation of grazing had a larger negative impact on rare taxa compared to common and widespread taxa (figure 4). For most groups of soil organisms and plants, rare species decreased more than the common species as a result of grazing removal, although for mites and bacteria this trend was not significant. For plants and nematodes, the diversity of rare species decreased more strongly (response ratio < −1) than for bacteria, fungi, protists, springtails and mites (response ratio between −0.1 and −0.6 (figure 4).

## 4. Discussion

**Figure 5.**
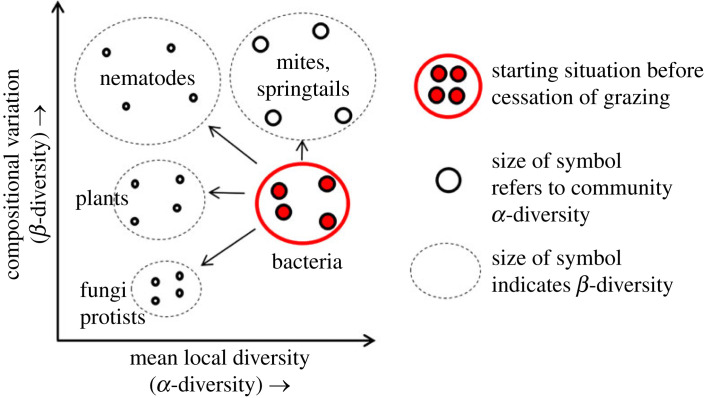
Long-term effects of cessation of grazing on local (*α*) and compositional (*β*) diversity. For all groups except mites, collembolans and bacteria, we found a significant decrease in species richness when grazers were excluded. There was a more varied response for *β*-diversity: some groups, such as nematodes, exhibited a strong community divergence, indicating that cessation of grazing results in increased *β*-diversity. Other groups, such as, fungi and protozoa exhibited a community convergence, indicating that cessation of grazing led to a decreased *β*-diversity, while the *β*-diversity of bacteria was not affected.

Results from this large-scale study including 12 montane grassland sites along an 800 km north–south latitudinal gradient across the UK shows consistent patterns in the response of *α*-diversity of soil microbial and soil fauna groups to cessation of grazing. Key soil fauna and microbial groups, with the exception of soil bacteria, showed marked declines in *α*-diversity following the cessation of livestock grazing, which also coincided with a marked decline in local plant species richness (figure 5). By contrast, the response of *β*-diversity to the cessation of grazing varied between groups of soil organisms (figure 5). While most soil microbial communities exhibited increased homogenization after cessation of grazing, we observed decreased homogenization for soil fauna after cessation of grazing. Although these patterns are consistent between different groups of soil organisms, and cessation of grazing ultimately shapes these changes in diversity, the exact mechanisms are less clear, and are further discussed below.

The decline in plant species richness with grazer exclusion is consistent with previous studies showing that extensive grazing generally has a positive effect on local plant diversity [12,48,49]. However, we found no evidence that plant species richness itself was a prominent proximate driver of changes in below-ground α-diversity. Rather, our analysis showed that other factors, especially soil pH, carbon concentration, soil moisture, bulk density, litter biomass and above-ground biomass, were the most important determinants of grazer exclusion-induced changes in below-ground α-diversity, largely consistent with previous studies showing that habitat characteristics are important determinants for the effect of grazer exclusion on soil communities in grasslands (e.g. [12,13,29,30,50]. Nevertheless, we speculate that, although plant communities were not identified as a direct driver of soil fauna communities, changes in plant communities may be one of the ultimate drivers underlying the observed patterns in α-diversity. For example, we observed a shift towards fern- or dwarf shrub-dominated vegetation in many of the abandoned plots on acid soils (electronic supplementary material, figure S1), which is often associated with reduced soil pH and increased litter mass [51]. Such vegetation-induced changes in soil biotic and abiotic conditions would then be the proximate driver of the observed changes in soil communities. This suggests that, rather than changes in plant species diversity or vegetation properties *per se*, shifts in plant species composition may ultimately drive the observed patterns in local below-ground richness.

At the local scale, effects of cessation of grazing on below-ground species were even more pronounced for relatively rare than for relatively widespread and common species. These results are consistent with McKinney & Lockwood's original (1999) idea of ‘biotic homogenization’ [10] (scenarios A and F in figure 1): relatively rare species suffer more from land use change than relatively common or widespread species. We speculate that a mix of drivers may be responsible for this pattern. First, an important driver may be the loss of certain (rare) plant species from the areas where grazing was halted. Different plant species are known to selectively influence community composition in their rhizosphere [52,53]. For example, most short grass species (e.g. *Cynosurus cristatus*, *Agrostis stolonifera*), legumes (e.g. *Trifolium repens*) and short herbs (e.g. *Bunium bulbocastanum*) decreased strongly or disappeared altogether after grazing exclusion. In addition, the selective loss of relatively rare species might result from a decrease in local heterogeneity after grazing exclusion as a result of a lack of small-scale trampling [54] or a lack of local defecation [55]. Because of a lack of ecological information about specific plant–microbe and plant–fauna relationships and changes in patterns of local heterogeneity, it is impossible to provide conclusive evidence for each of the two hypotheses. As both processes typically coincide with removal of grazing [9,56], it is likely that the resulting pattern can be generalized to other systems: cessation of grazing results in the loss of below-ground species richness through the combined effect of a loss of local heterogeneity and local plant species richness.

### (a) Drivers of below-ground *β*-diversity

In grasslands, extensive grazing generally leads to spatial heterogeneity in above-ground vegetation [9] where different patches represent different phases on a successional gradient [57]. Cessation of grazing thereby becomes a homogenizing factor that pushes patches towards a climax stage of generally lower above-ground *β*-diversity [48]. As a result, one might expect below-ground *β*-diversity to exhibit a similar decrease in response to cessation of grazing (i.e. greater homogenization in community composition). However, in contrast to the consistent negative responses for *α*-diversity, our results show remarkably mixed responses for *β*-diversity of different groups of below-ground biota. We observed a strong decline in *β*-diversity for eukaryotic soil microbes (fungi, protists), no change in *β*-diversity of prokaryotic soil microbes, and an in increase in *β*-diversity for springtails, mites and nematodes. These differences in response between larger bodied and smaller bodied soil organisms may result from differences in the sensitivity of these groups of organisms to shifts in plant species composition and/or changes in soil physical parameters that happen as a result of grazer removal. A plethora of studies has shown that soil microbial community composition is strongly related to the composition of the plant species community [52,58–60], as many microbial taxa are directly dependent on carbon sources (e.g. exudates and litter) from plants. Soil animals are also ultimately dependent on plant-derived carbon, but, due to their greater mobility and size, may also be affected by other environmental factors that change in response to the cessation of grazing. We therefore propose that the changes in vegetation community and the accompanying changes in root exudation patterns, litter quality, local changes in pH gradients [61,62] as well as variation in litter recalcitrance [63] may explain the observed differences in *β*-diversity for soil microbial groups, whereas the observed physical differences in soil properties, soil organic matter and soil structure, and the spatial variation therein might be more important for the spatial distribution larger bodied species [64,65].

A last remaining question is why *β*-diversity of soil fauna actually *increases* in response to cessation of grazing. Here, we speculate that this might be due to increased cover and patch size of mid-late successional (clonal) plant species. Indeed, clonal plant species characteristic of mid to late successional stages (e.g. *C. vulgaris*, *Pteridium aquilium, Molinia caerulea* and *D. cespitosa*) were more abundant in the abandoned plots. These species are generally associated with more complex food webs and a greater abundance of higher trophic levels as a result of larger below-ground carbon inputs [66]. Patches that consist of different functional groups (heather species, legumes, grasses, other shrubs, ferns, mosses) can differentially affect the diversity of organisms through changes in resource supply and other abiotic properties [67,68]. Evidence for the idea that such patches are associated with different soil communities comes from vegetation removal experiments, which show that removal of entire functional groups has major effects on below-ground species composition and functioning [69]. In our study sites, this increasing patch size is exemplified by the replacement of species rich grasslands dominated by short grasses and herbs by clonal growth of patch forming species such as ferns (e.g. *P. aquilinum*), dwarf shrubs (e.g. *C. vulgaris, V. myrtillus, E. tetralix*) and tall grasses (e.g. *M. caerulea* and *D. cespitosa*). This change in patchiness in the vegetation after cessation of grazing may thus result in increased medium-large scale spatial heterogeneity. We hypothesize that the resulting divergence in below-ground communities may start once the lack of grazing permits these clonal structures to become locally dominant, although more rigorous experiments are needed to test the relative importance of these different possible mechanisms, particularly on the sequence and hierarchy of effects.

## 5. Conclusion

By analysing a comprehensive dataset of key below-ground taxa, we show that cessation of grazing on montane grasslands with a long history of extensive sheep grazing leads to significant declines in *α*-diversity of soil organisms, while *β*-diversity of soil fauna and soil microbes show a contrasting response. This illustrates that extensive grazing plays a key a role in regulating biodiversity of below-ground communities, and highlights that the cessation of livestock grazing can result in a range of deleterious effects, much in line with recent work on above-ground invertebrate communities [70]. However, the exact mechanisms and processes generating these patterns remain poorly understood and warrant further investigation. Large swaths of Europe are currently being subjected to ‘rewilding’, an approach to nature conservation that involves the combination of cessation of historic livestock grazing and replacement by (often low densities of) wild ungulates and/or other grazers, including rare cattle breeds and horses. Our results suggest that such a ‘rewilding’ approach to nature conservation involving the complete removal of domestic livestock might not lead to an associated increase in the diversity of below-ground organisms, at least not in the areas where wild herbivores or other grazers are not reintroduced. Rather, given that current densities of natural grazers and browsers in historically grazed systems are low, particularly compared to previous interglacial periods [71], we expect profound negative impacts of instantaneous cessation of grazing on below-ground diversity. This suggest that a gradual reduction of extensive grassland management needs to be accompanied by a gradual increase of natural grazers when aiming to conserve below-ground biodiversity.

## Data Availability

The data are provided in electronic supplementary material [72].
